# Supplementary material for: Atomically ordered non-precious Co3Ta intermetallic nanoparticles as high-performance catalysts for hydrazine electrooxidation
Source: Nat Commun. 2019 Oct 4;10:4514. doi: 10.1038/s41467-019-12509-7 (PMC6778194; doi:10.1038/s41467-019-12509-7)
Supplement: Supplementary file 1 — Supporting Information [file 41467_2019_12509_MOESM1_ESM.pdf]

Supporting Information

Atomically ordered non-precious Co_3Ta intermetallic nanoparticles as high-performance catalysts for hydrazine electrooxidation

Feng et al.

Supplementary Figures:

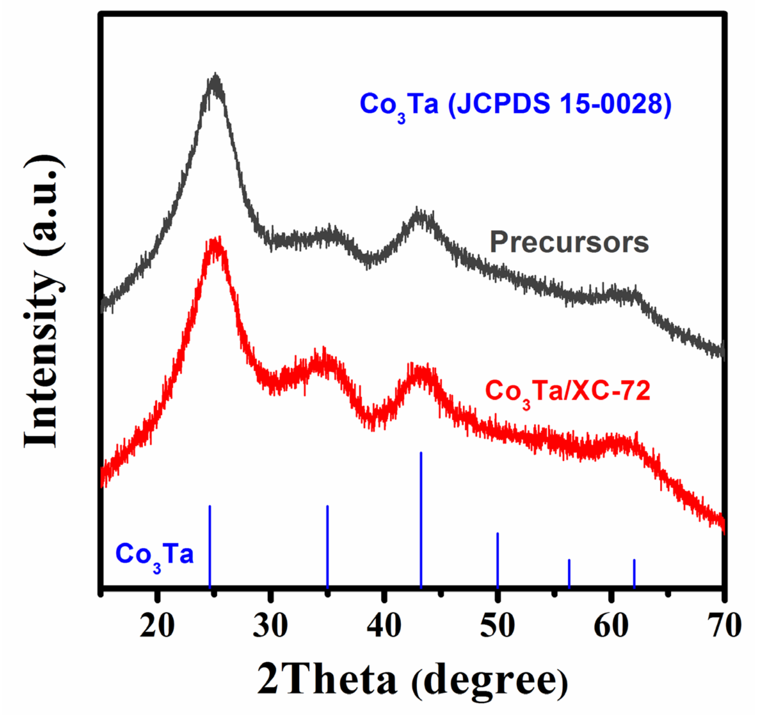

Supplementary Figure 1. The Powder XRD patterns of Co_3Ta/C intermetallic nanoparticles and precursors. The lines at the base show the standard XRD pattern of Co_3Ta (JCPDS 15-0028).

After the co-reduction of tantalum and cobalt salts in mixed solution, the filting was washed with rigorously dried and degassed THF and hexanes. The cleaned filting was annealed in a H_2/Ar flow at 300℃ for 3h, and then washed with argon-saturated ultrapure water. The as-obtained products were marked as precursors.

Supplementary Table 1. A list of the reported early-transition-metal (III-VB transition metal) intermetallic compounds.

Nano-Intermetallic Compounds	Particle Size (nm)	Calcination Temperature (℃)	Electrocatalysis	Noble/Non-noble	Reference
Pt_3Ti	14-60	600	Formic acid and methanol oxidation reaction	Noble	1
Pt_3Ti Pt_3V	3-9	700 650	Methanol oxidation reaction	Noble	2
Pt_3Zr	~100	1000	Formic acid and ethanol oxidation reaction	Noble	3
Pt_3Nb	<150	1000	Ethanol oxidation reaction	Noble	4
Pt_3Ta	<100	1000	Ethanol oxidation reaction	Noble	5
Pt_3Y Pt_3Sc Pt_3Lu	5-20	650	/	Noble	6
Co_3Ta	3-10	400	Hydrazine oxidation reaction	Non-noble	This work

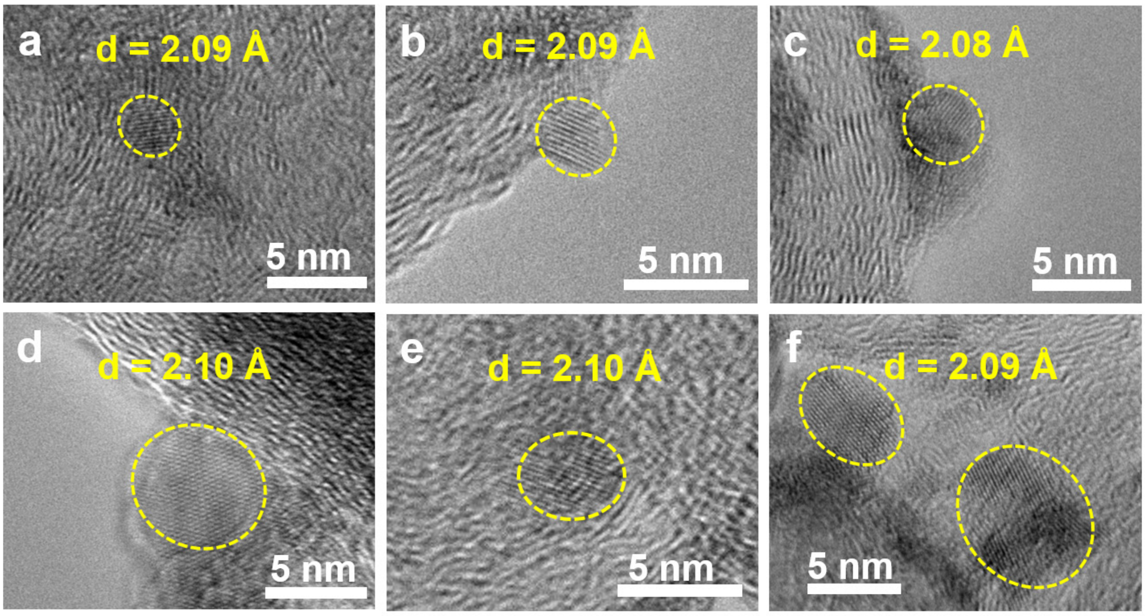

Supplementary Figure 2. The crystal lattice images of different Co_3Ta intermetallic nanoparticles.

The different Co_3Ta nanoparticles were selected to observe the lattice spacings, and the obtained lattice spacings are averaged at 0.208 and 0.210 nm, corresponding to the (111) plane of Co_3Ta intermetallic compounds.

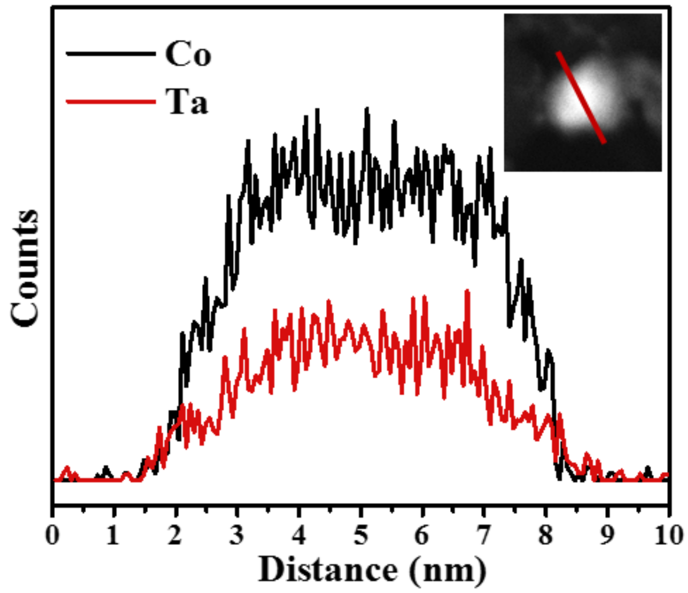

Supplementary Figure 3. Line scanning profiles of single Co_3Ta nanoparticle, indicating both Co and Ta homogeneously disperse throughout the nanoparticle.

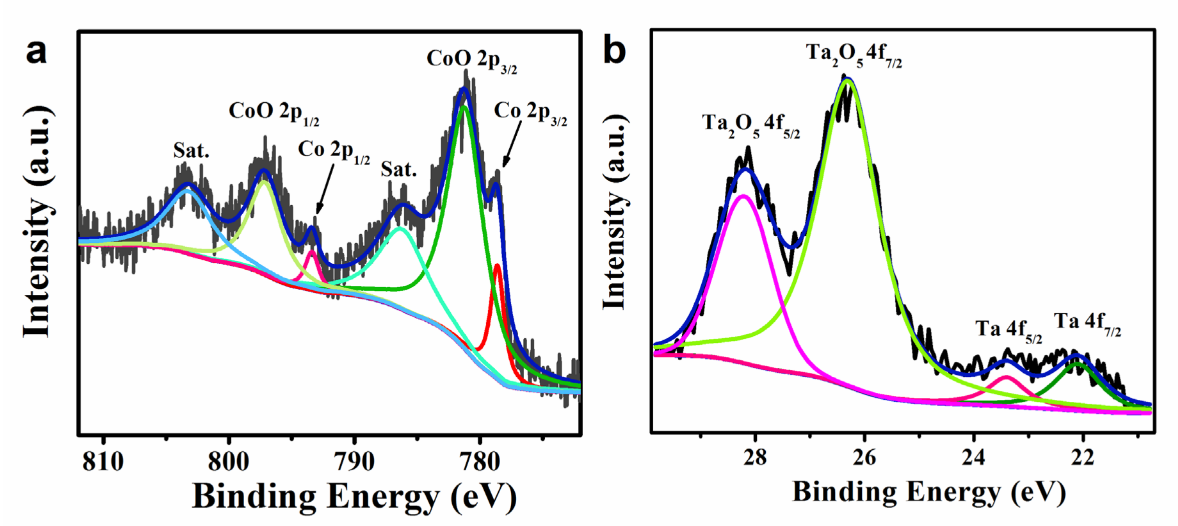

Supplementary Figure 4. XPS spectrums of Co_3Ta/C nanoparticles. **a** Co 2p XPS spectrum. **b** Ta 4f XPS spectrum. Because of the extremely oxyphilic nature, the samples should be isolated from oxygen as much as possible.

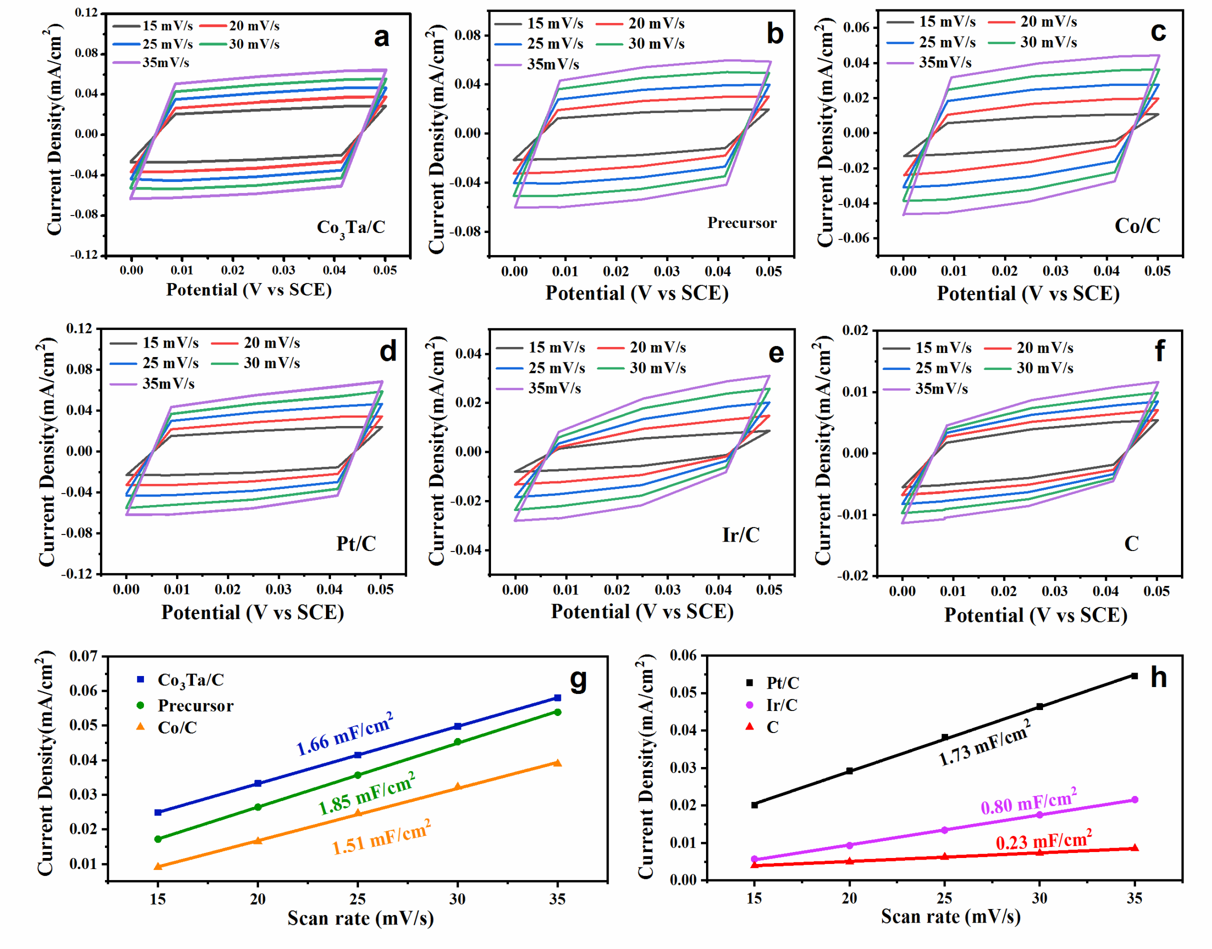

Supplementary Figure 5. The electrochemical double-layer capacitance (EDLC) measurements of (**a**) Co_3Ta/C, (**b**) Precursor, (**c**) Co/C, (**d**) Pt/C, (**e**) Ir/C, and (**f**) C. (**g** and **h**) The corresponding current density-scan rates curves of the above samples.

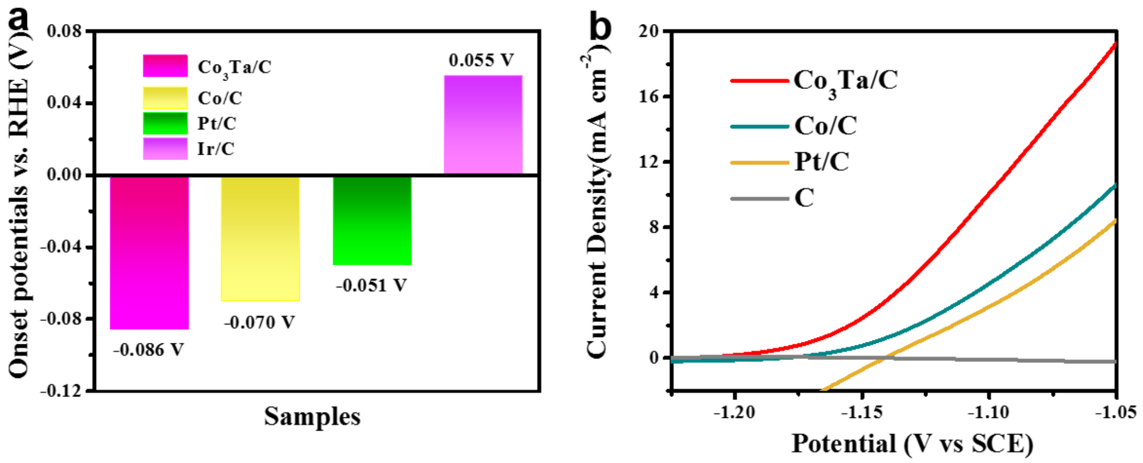

Supplementary Figure 6. a Comparisons of the onset potentials (E_{on}) of Co_3Ta/C, Co/C, commercial Pt/C, and commercial Ir/C (vs. RHE). **b** LSV curves of Co_3Ta/C, Co/C, commercial Pt/C, and commercial Ir/C (vs. SCE), showing Co_3Ta/C has the lowest E_{on} and highest intrinsic activity toward HzOR.

Here, in order to compare other onset potential reported by previous works, the onset potentials (both vs. RHE and SCE) of our samples are provided.

Supplementary Table 2. A comparison of onset potentials for hydrazine oxidation towards different catalysts.

Catalysts	Concentration of N_2H_4 (M)	Electrolyte	Scan Rate (mV s^{-1})	Onset Potential (V)	Ref.
NiFe-LDH/Ni Foam	2	1 M KOH	100	-0.2 (Vs. SCE)	7
Cu Film/Cu Foam	0.2	3 M NaOH	5	-0.78(Vs. SCE)	8
$Ni_{0.6}Co_{0.4}$ Nanosheets/Ni Foam	0.5	3 M KOH	5	-1.13(Vs. SCE)	9
CoNi-S Nanosheets/Ni Foil	0.02	0.1 M KOH	10	0.1 (Vs. RHE)	10
Ni Nanosheets/Ni Foam	0.5	3 M KOH	5	-0.05(Vs. RHE)	11
Ni_2P Nanosheets/Ni Foam	0.5	1 M KOH	5	-0.05(Vs. RHE)	12
$CoSe_2$ nanosheets/Ni Foam	0.5	1 M KOH	5	-0.045(Vs. RHE)	13
Fe-CoS_2 Nanosheets	0.1	1 M KOH	5	0 (Vs. RHE)	14
$Pt_{0.2}Ni_{0.8}$/C	0.5	0.1 M PBS	10	0.03(Vs. RHE)	15
N- and S-co-doped Carbon	0.05	0.1 M PBS	10	0.38(Vs. RHE)	16
Co_3Ta/C	0.2	3M KOH	5	-1.175(Vs. SCE)<br>-0.086(Vs. RHE)	This work

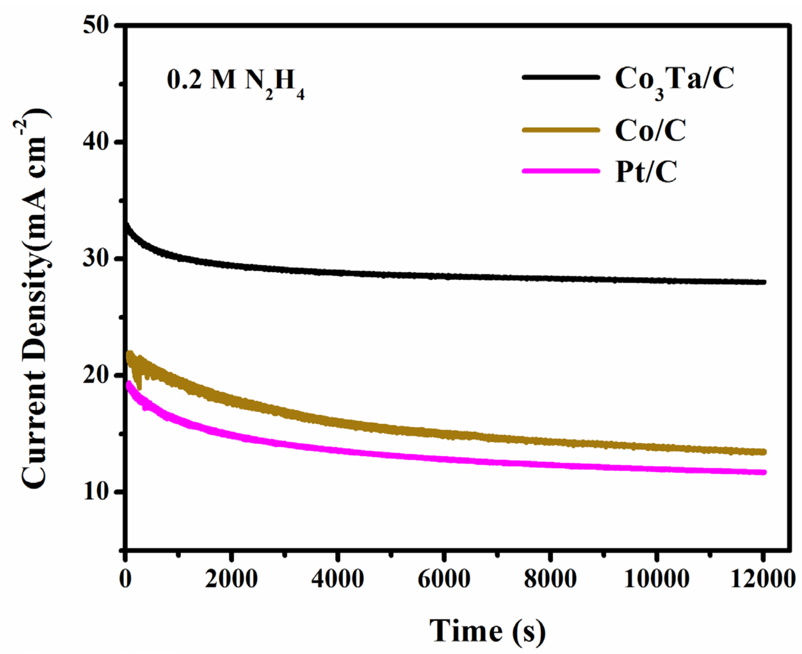

Supplementary Figure 7. Stability testing for 12000 s of the Co_3Ta/C, Co/C, and commercial Pt/C, demonstrating Co_3Ta/C nanoparticles an excellent stability.

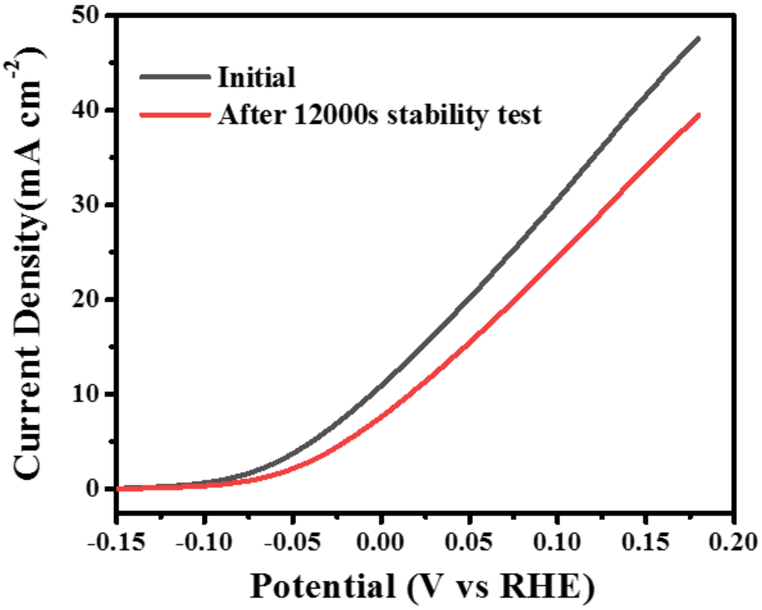

Supplementary Figure 8. LSV curves of Co_3Ta/C nanoparticle before and after stability test, showing only a 10 mV positive shift.

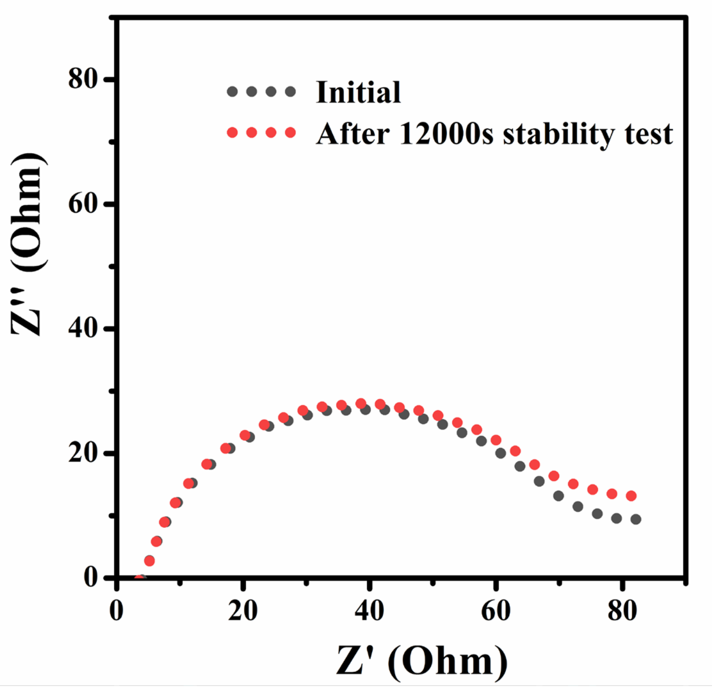

Supplementary Figure 9. The Nyquist plots of Co_3Ta/C nanoparticle before and after stability test, showing a negligible change.

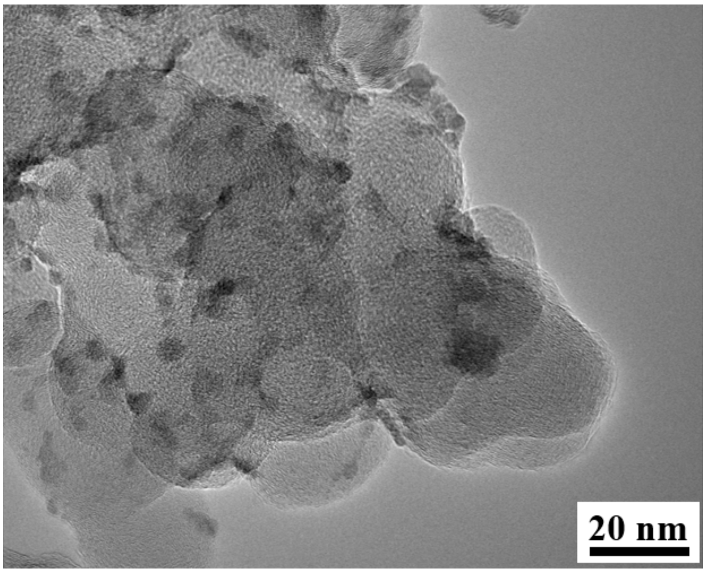

Supplementary Figure 10. TEM image of Co_3Ta/C nanoparticles after stability test, showing no obvious damage and particle agglomerations.

Supplementary Table 3. The net charge of Ta and Co in Co_3Ta from Bader charge analysis. Co gain the some electrons from Ta, showing the tuning of electronic structure of the Ta atom by Co. which is consistent with the analytical results of XAFS.

Element	Net Charge (e/atom)
Ta	1.08
Co-I	-0.23
Co-II	-0.36
Co-III	-0.49

Generally speaking, the properties of chemical compound and materials are described in charge transfer between atoms, but the atomic charge in molecules or solids are not physical observables and it is not defined by quantum mechanical theory. There are many different computational schemes about atomic charge in molecular and solid have been proposed and the barder charge method is most common in solid calculation. In order to determine the charge transfer between Co and Ta in Co_3Ta, the bader charge of this ordered intermetallic structure was calculated. Firstly, the total charge density of Co_3Ta bulk was calculated by using DFT method, then the bader charge software developed by Henkelman group (http://theory.cm.utexas.edu/henkelman/code/bader/) was employed and the bader charge results was listed in Supplementary Table 3. It was confirmed the charge transfer between Co and Ta in Co_3Ta. As shown in Supplementary Table 3, Co gain the some electrons from Ta, showing the tuning of electronic structure of the Ta atom by Co. which is consistent with the analytical results of XAFS.

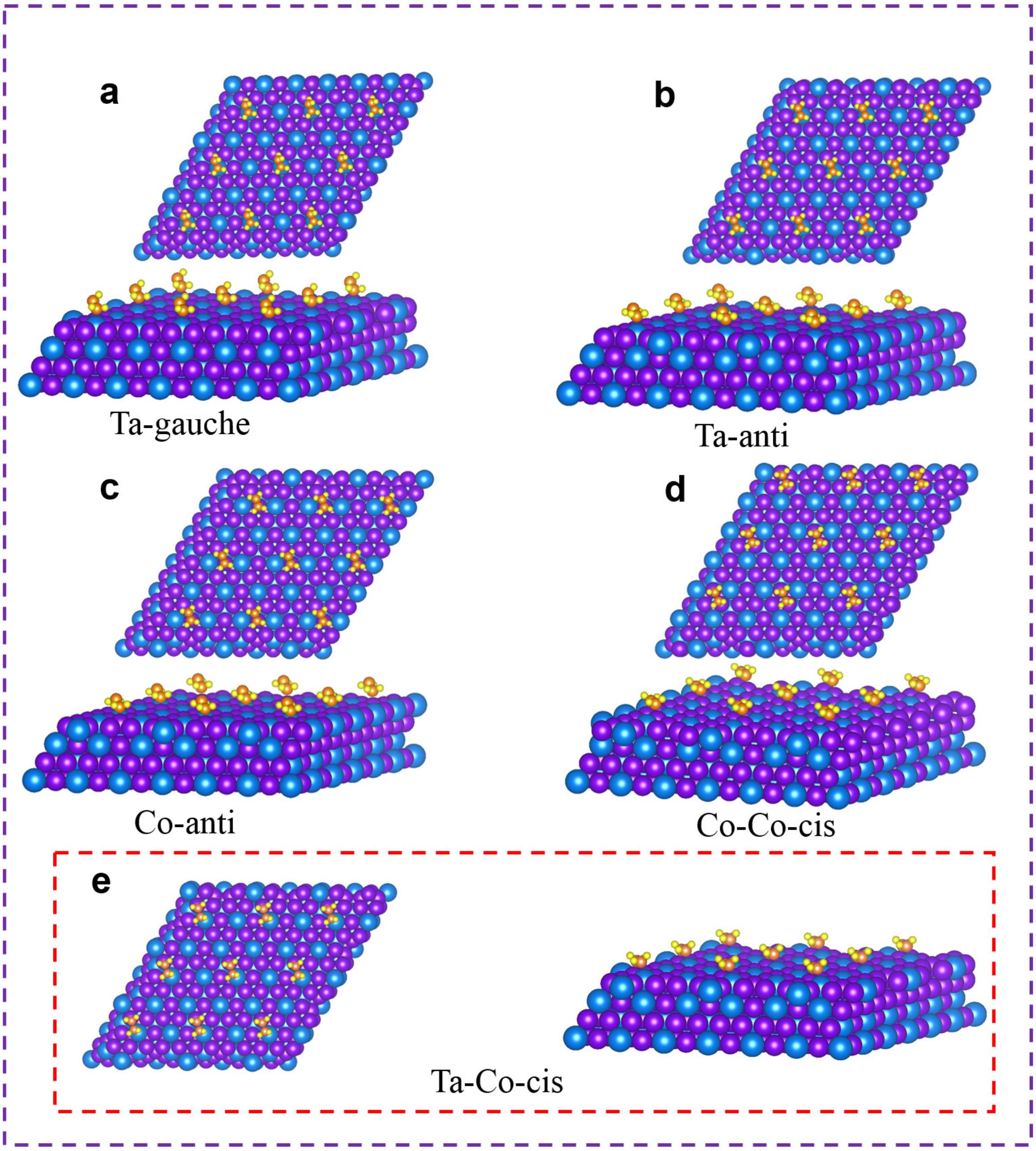

Supplementary Figure 11. The top and side views of different adsorption conformations of N_2H_4 molecular on Co_3Ta (111) surface. **a** Ta-gauche. **b** Ta-anti. **c** Co-anti. **d** Co-Co-cis. **e** Ta-Co-cis. The conformation of Ta-Co-cis is selected as the adsorption conformation, which is the most stable one.

Supplementary Table 4. Adsorption energies of different adsorption conformations of N_2H_4 molecular on Co_3Ta (111) surface. The conformation of Ta-Co-cis is the most stable adsorption conformation.

Conformations	E_a (eV)
Ta-gauche	-0.07
Ta-anti	-0.14
Ta-Ta-cis	Inexistence
Co-gauche	Unstabitily
Co-anti	0.25
Co-Co-cis	0.22
Ta-Co-cis	-0.25

Supplementary Table 5. The surface energies of different miller index surfaces of Co_3Ta, indicating the (111) plane is the most stable one.

Surface type	Surface Energy (eV/Å^2)
(100)	0.175
(110)	0.174
(111)	0.155
(210)	0.182
(211)	0.177

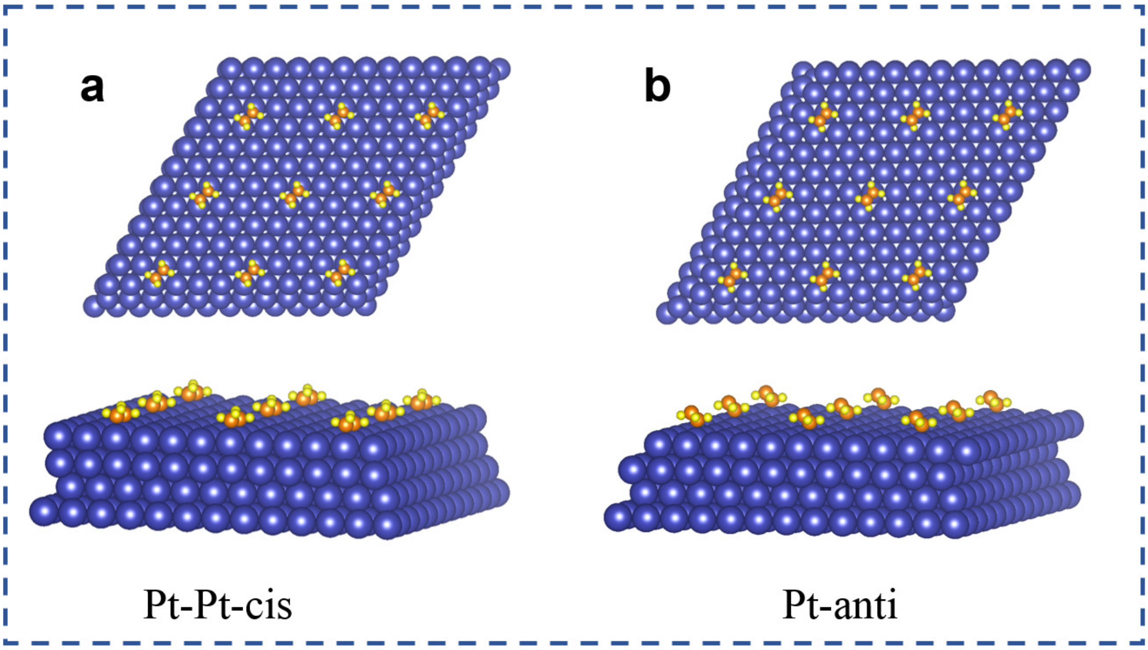

Supplementary Figure 12. The top and side views of two adsorption conformations of N_2H_4 molecular on Pt (111) surface. **a** Pt-Pt-cis. **b** Pt-anti. The conformation of Pt-anti is selected as the adsorption conformation, which is the more stable one.

Supplementary Table 6. Adsorption energies of two adsorption conformations of N_2H_4 molecular on Pt (111) surface. The conformation of Pt-anti is more stable.

Conformations	E_a (eV)
Pt-gauche	Unstabitily
Pt-anti	-1.15
Pt-Pt-cis	-0.92

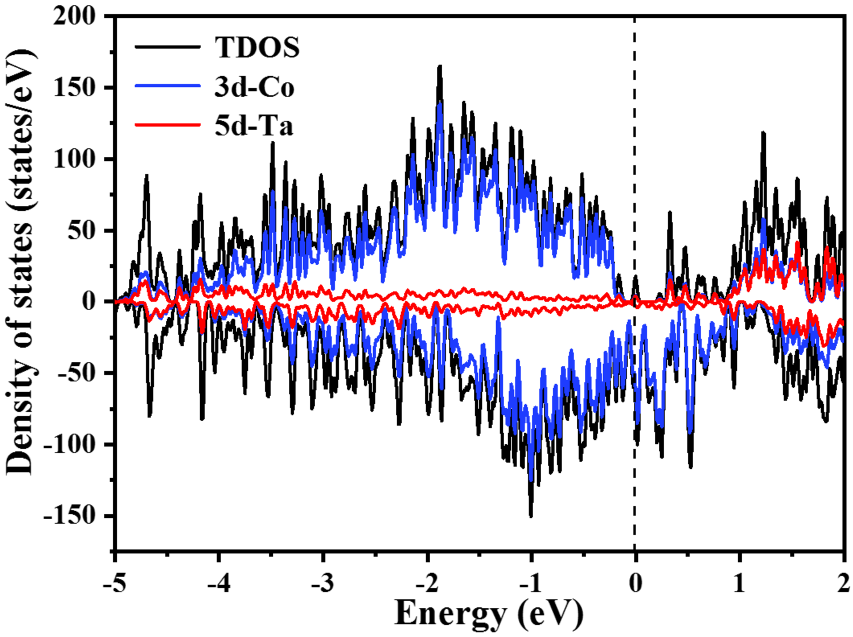

Supplementary Figure 13. The DOS curves of Co_3Ta (111) surface, demonstrating that the electron structure of the surface is similar to bulk metal. Because of the magnetic property of Co, the spin down parts is larger than spin up parts in the near of Fermi energy level.

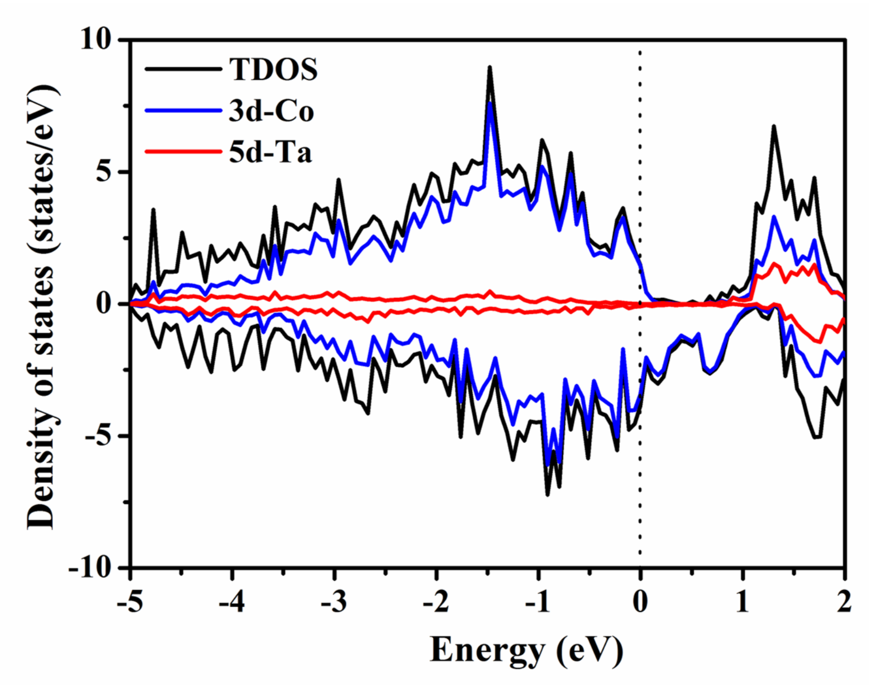

Supplementary Figure 14. The DOS curves of bulk Co_3Ta.

Supplementary Table 7. The relationship between the adsorption energy (E_{ads}) and number of layers. The results show that the adsorption energy with 4 layers thickness is similar to that with 6 layers and 8 layers.

Layers	$E(N_2H_4\&surface)$/eV	E(surface)/eV	$E(N_2H_4)$/eV	E_{ads}/eV
2_layers	-91.58984268	-60.62858533	-30.29083739	-0.67041996
4_layers	-159.0308869	-128.0005605	-30.29083739	-0.73948901
6_layers	-226.6693516	-195.6227054	-30.29083739	-0.75580885
8_layers	-294.2978521	-263.2682775	-30.29083739	-0.73873719

Supplementary Table 8. The results of ΔE, ΔZPE and TΔS for Co_3Ta (111) and Pt (111).

Reaction steps	Surface	ΔE (eV)	ΔZPE (eV)	TΔS (eV)
$N_2H_4* \rightarrow N_2H_3*+H^++e$	Co_3Ta(111)	-0.59	-0.12	-0.06
	Pt(111)	-0.31	-0.11	-0.04
$N_2H_4* \rightarrow N_2H_2*+2H^++2e$	Co_3Ta(111)	-0.17	-0.33	0.12
	Pt(111)	0.39	-0.32	0.14
$N_2H_4* \rightarrow N_2H*+3H^++3e$	Co_3Ta(111)	-0.01	-0.51	0.30
	Pt(111)	0.54	-0.52	0.32
$N_2H_4* \rightarrow N_2*+4H^++4e$	Co_3Ta(111)	-0.60	-0.68	0.55
	Pt(111)	0.01	-0.70	0.55

Supplementary References

1. Abe, H. et al. Electrocatalytic performance of fuel oxidation by Pt_3Ti nanoparticles. *J. Am. Chem. Soc.* **130,** 5452-5458 (2008).
2. Cui, Z. et al. Synthesis of structurally ordered Pt_3Ti and Pt_3V nanoparticles as methanol oxidation catalysts. *J. Am. Chem. Soc.* **136**, 10206-10209 (2014).
3. Ramesh, G. V. et al. Stimulation of electro-oxidation catalysis by bulk-structural transformation in intermetallic $ZrPt_3$ nanoparticles. *ACS Appl Mater Interfaces* **6**, 16124-16130 (2014).
4. Ramesh, G. V. et al. $NbPt_3$ intermetallic nanoparticles: highly stable and CO-tolerant electrocatalyst for fuel oxidation. *ChemElectroChem*. **1**, 728-732 (2014).
5. Kodiyath, R. et al. Promoted C-C bond cleavage over intermetallic $TaPt_3$ catalyst toward low-temperature energy extraction from ethanol. *Energy Environ. Sci.* **8**, 1685-1689 (2015).
6. Kanady, J. S. et al. Synthesis of Pt_3Y and other early-Late intermetallic nanoparticles by way of a molten reducing agent. *J. Am. Chem. Soc.* **139**, 5672-5675 (2017).
7. Li, Z. et al. Fast electrosynthesis of Fe-containing layered double hydroxide arrays toward highly efficient electrocatalytic oxidation reactions. *Chem Sci* **6**, 6624-6631 (2015).
8. Lu, Z. et al. Superaerophobic electrodes for direct hydrazine fuel cells. *Adv. Mater.* **27**, 2361-2366 (2015).
9. Feng, G. et al. Single crystalline ultrathin nickel-cobalt alloy nanosheets array for direct hydrazine fuel cells. *Adv. Sci.* **4**, 1600179 (2017).
10. Zhou, L. et al. Hierarchical CoNi-Sulfide nanosheet arrays derived from layered double hydroxides toward efficient hydrazine electrooxidation. *Adv. Mater.* **29**, 1604080 (2017).
11. Kuang, Y. et al. Single-crystalline ultrathin nickel nanosheets array from in situ topotactic reduction for active and stable electrocatalysis. *Angew. Chem. Int. Ed.* **55**, 693-697 (2016).
12. Tang, C. et al. Energy-Saving Electrolytic Hydrogen Generation: Ni_2P Nanoarray as a High-Performance Non-Noble-Metal Electrocatalyst. *Angew. Chem. Int. Ed.* **56**, 842-846 (2017).
13. Zhang, J. et al. Anodic Hydrazine Oxidation Assists Energy-Efficient Hydrogen Evolution over a Bifunctional Cobalt Perselenide Nanosheet Electrode. *Angew. Chem. Int. Ed.* **57**, 7649-7653 (2018).
14. Liu, X. et al. Self-powered H_2 production with bifunctional hydrazine as sole consumable. *Nat. Commun.* **9**, 4365 (2018).

15. Wang, J. et al. A bifunctional catalyst for efficient dehydrogenation and electro-oxidation of hydrazine. *J. Mater. Chem. A.* **6**, 18050-18056 (2018).
16. Cazetta, A. L. et al. Bone char-derived metal-free N- and S-co-doped nanoporous carbon and its efficient electrocatalytic activity for hydrazine oxidation. *Appl. Catal., B: Environ.* **225**, 30-39 (2018).
